# Level of implementation of digital transformation in dental radiology centers in Metropolitan Lima: A cross-sectional study

**DOI:** 10.4317/jced.62023

**Published:** 2024-09-01

**Authors:** Elmer Flores-Leiva, Veralucía Milagros Guardia-Chocce, Luis Ernesto Arriola-Guillén

**Affiliations:** 1 DDS, MS. Professor of the Division of Orthodontics School of Dentistry, and graduate of the specialty of Oral and Maxillofacial Radiology, Universidad Científica del Sur, Lima, Perú; 2 DDS graduate of the specialty of Oral and Maxillofacial Radiology, Universidad Científica del Sur, Lima, Perú; 3 Ph.D. and Associate Professor of the Division of Orthodontics, School of Dentistry, Universidad Científica del Sur, Lima, Perú

## Abstract

**Background:**

Digital transformation (DT) involves introducing digital technologies into business models in all areas. This research aimed to evaluate the level of implementation of DT using the digital health indicator in private radiology centers in Lima, Perú.

**Material and Methods:**

Forty-five randomly selected radiology centers included in a database of 50 registered centers were evaluated. The inclusion criteria were having a web domain and institutional email address. They were digitally surveyed using a digital survey (HIMSS DHI Rapid) measuring four dimensions: interoperability, governance and workforce, predictive analytics, and person-enabled health. These indicators allowed determination of the digital transformation level of a radiological company. The level of implementation was measured quantitatively on a scale from 0 to 400, and the Kruskal Wallis test (p>0.05) was used to compare DT according to the geographical location of the centers.

**Results:**

The digital health indicator obtained was 60.24 ± 43.14 out of 400 achievable points. The dimensional analysis in terms of interoperability was 24 ± 18.09, followed by governance and workforce at 23.44 ± 18.58, person-enabled health at 18.73 ± 15.63, and finally, predictive analysis at 16.18 ± 13.51. No significant differences were found in health indicator dimensions according to the geographical location (*p*>0.05).

**Conclusions:**

DT in maxillofacial radiology centers in Lima is at an initial level. Radiology centers should take this situation into account to have relevant information for making diagnostic and treatment decisions and to provide better preventive health policies to benefit the population.

** Key words:**Digital transformation, digital health indicator, dental radiology.

## Introduction

Digital transformation (DT) is a new area of research for companies. It involves incorporating digital technologies into business models across all areas, leading to significant changes in how organizations operate and deliver value to customers (1-5). The integration of digital technologies allows the creation of new methods for delivering value. As a result, this necessitates a transformation in the culture, processes, structures, and strategies of an organization (6-9).

As the health sector is one of the most complex organizations, DT could contribute enormously to optimizing clinical and administrative processes and generate good experience for all those involved (patients, healthcare workers, and administrative staff). Although DT is challenging, the new way of working would greatly facilitate the daily work experience, considering that its implementation would involve all organization members (10-13). In a joint statement about DT in the health sector, the World Health Organization and the Pan American Health Organization published the eight guiding principles for DT, which included: 1.- Universal connectivity 2.- Digital goods 3.- Inclusive digital health 4.- Interoperability 5.- Human rights 6. Artificial intelligence 7.- Information security and 8.- Public Health Architecture (14-16).

Many radiology centers have recently faced limitations due to decreased dental care, especially during the COVID-19 pandemic. A study conducted in Ecuador revealed that while most centers attempted to enhance their sales through digital media channels such as websites, social media, and search engines, the internal processes of radiology centers remained traditional. The adoption of digital technologies was mainly considered as a protective measure to prevent the insolvency of radiology centers rather than an integral part of their organizational strategy (17,18).

All cities need to monitor the progress of their DT processes to establish improvement strategies. In Peru, there have been no research studies evaluating DT in radiology centers, except for one study conducted in dental offices in the district of Miraflores in Lima. This study revealed limited openness to DT due to the limited knowledge of the administrative staff regarding the benefits and positive influence of DT, thereby hindering the implementation of DT as part of the business plan (19).

Three experts have validated a scale for measuring DT, which includes the digital health indicator (DHI), a tool known worldwide for assessing the level of DT in health sector institutions (19,20). One study in Australia used the DHI to assess digital health capacity and identified areas for improvement and recognized strengths and weaknesses to implement a better strategy. The maximum score of the DHI is 400 points, with an average score of 143 points found in Oceania. However, private centers in other countries have described even lower scores (21,22).

The rapid advancement of digital technology has significantly impacted various aspects of our daily lives, such as television, banking, and online shopping. However, dentistry continues to rely on traditional processes, including manual appointment scheduling and medical record-keeping. Integrating digital technology into dentistry is essential for enhancing the overall experience for both the institution and patients (18,23-25). Therefore, this study aimed to evaluate the level of implementation of DT using the DHI in private radiology centers in Lima, Perú.

## Material and Methods

The present study was approved by the Ethics and Research Committees of the Universidad Científica del Sur with approval code N° POS-117-2023-00562. This cross-sectional study was conducted in 45 radiological centers in metropolitan Lima included a database of 50 registered centers. It is worth mentioning that this number sample size was higher than the minimum required (39). The study employed a formula to estimate an average (mean value of DT) in a known population, with a level of 99%, using the free access program https://www.fisterra.com.

Subsequently, the selected radiology centers were administered the Healthcare Information and Management Systems Society (HIMSS) DT survey, which is developed and calculated digitally on the HIMSS website (HIMSS DHI Rapid), based on the following dimensions of the DHI.

1. Interoperability: This measure measures how the center functions operationally. It consists of three questions that evaluate the connection, security, and alert system level.

2. Governance and workforce: measures, in general, the administration of the policies, guidelines, and transparency, based on three questions.

3. People-enabled health: measures patient care, risk management, and user accessibility, with the use of three questions.

4. Predictive analysis measures data predictivity. It contains three questions about whether the center to be evaluated has data analysis tools.

The survey was conducted online using an electronic device and a web page. It included 12 questions to assess the level of implementation for each question. After completing the questionnaire, the evaluating institution requested the respondent’s institutional email and country and sent the report to the email provided for analysis and data processing.

The radiology centers included in the study were chosen based on specific criteria, such as offering panoramic radiographic services and having institutional email. In addition, the geographic location of the radiology centers was considered when assessing each aspect of DT.

-Statistical analysis 

The data obtained were entered into the statistical program SPSS version 27 (IBM SPSS, Chicago, Illinois, USA) and the results were expressed in graphs and Tables. Furthermore, the Kruskal-Wallis non-parametric test compared DT values according to geographical area (*P*<0.05).

## Results

**Figure 1:**
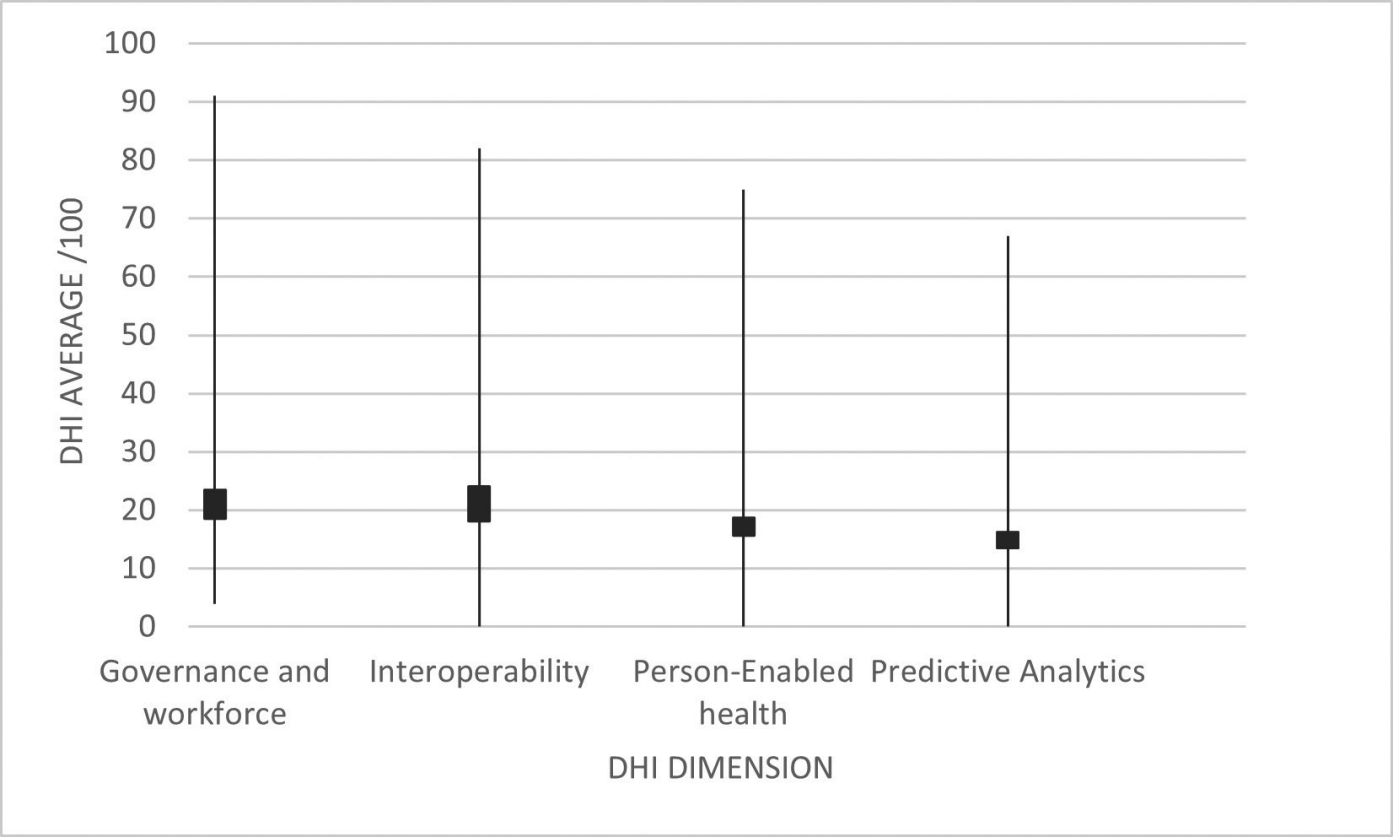
DHI dimension values of the 45 radiological centers in Metropolitan Lima.

The average DHI of the 45 radiological centers evaluated was 60.24 ± 43.14 out of a maximum of 400. The dimensional analysis of the DHI showed a mean interoperability of 24 ± 18.09, followed by governance and workforce with 23.44 ± 18.58, person-enabled health with 18.73 ± 15.63, and finally, predictive analytics with 16.18 ± 13.51 (Fig. 1).

**Figure 2:**
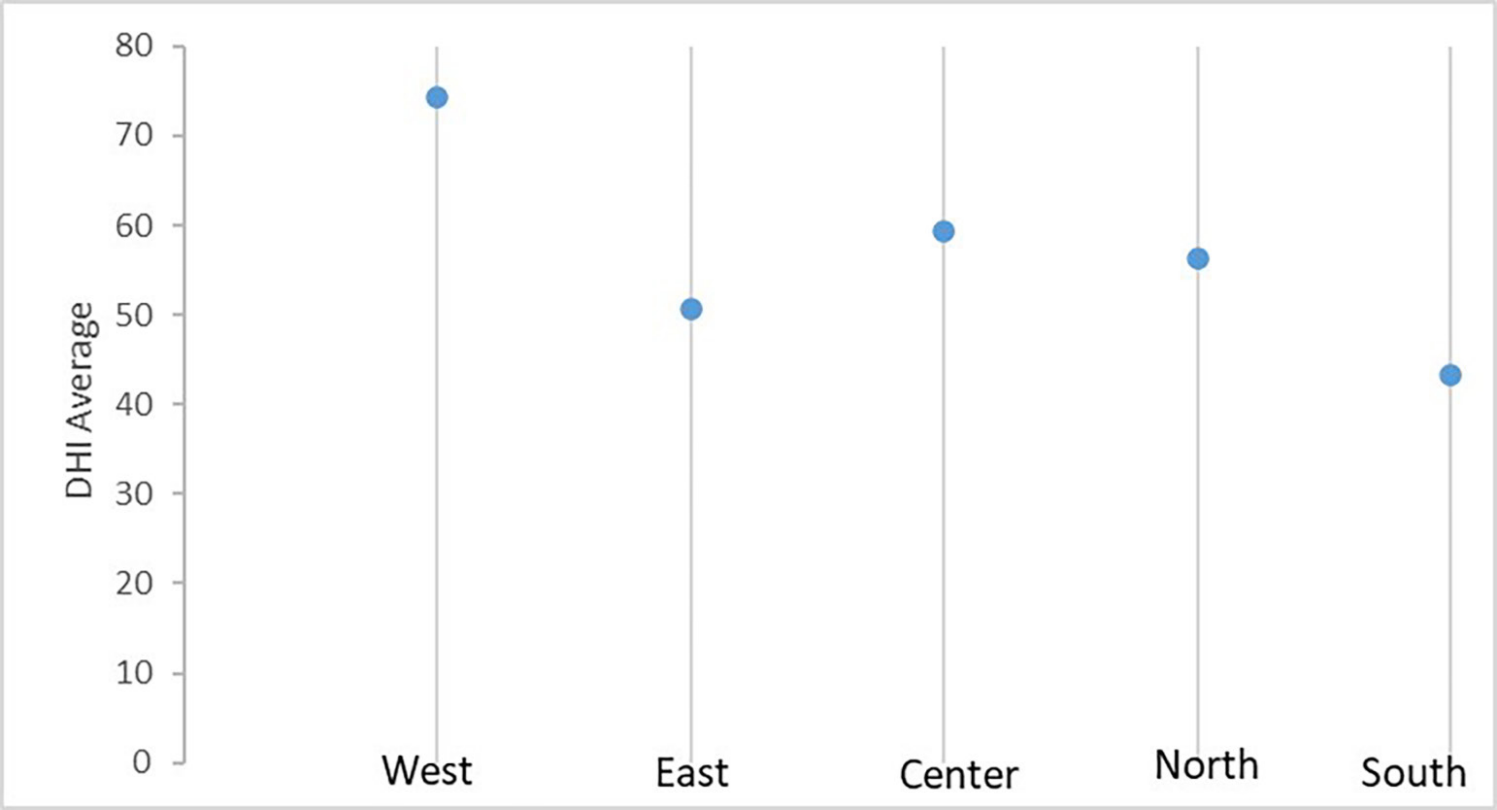
DHI values according to geographic location in different areas of Metropolitan Lima.

When the indicator of the radiological centers of Metropolitan Lima was measured according to geographic location and distributed by socioeconomic level, Western Lima showed the best health index, with a mean of 74.19 ±58.46 while Southern Lima had a lower index, with a mean of: 43.20±16.42, and the remaining areas of the city showed values similar to the mean (Fig. 2).

**Figure 3:**
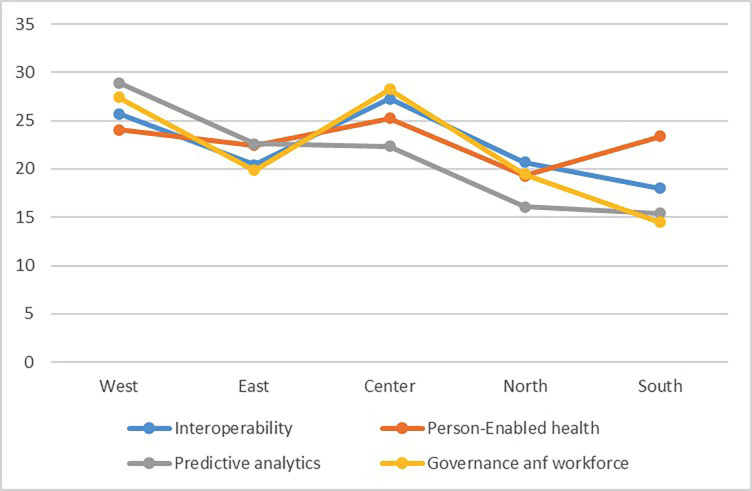
Representation of the dimensions according to Lima areas.

Similarly, the percentages of dimensions of the health indicator were measured based on the location and socioeconomic distribution in Metropolitan Lima: Interoperability; western Lima: 29.19 ± 23.58 and southern Lima: 16.6 ±10.41. Person-enabled health; western Lima: 21.19 ±20.31 and southern Lima: 17.09 ±9.9. Predictive analysis: western Lima 22.88 ± 16.65 and southern Lima 8.2 ± 4.97. Governance and labor force; western Lima: 30.13 ± 24.35 and southern Lima 13.6 ±10.41. The remaining areas of Lima presented values similar to the general dimensional mean (Fig. 3). These dimensions were compared according to geographic location using the Kruskal-Wallis test, and no significant difference were found in any of the comparisons ( Table 1).

## Discussion

According to the DHI index, DT in the radiology centers in Metropolitan Lima was low and less than 50% in an index with which the maximum score is 400 points. A study by Snowdon *et al*. (2020) (20) determined that for a health institution to be digitally transformed, it must achieve a score of 400. However, the score obtained by the radiology centers in our study was 60 points, which demonstrates that these health facilities are at a primary level of DT, despite being in the private sector and with technological equipment.

The dimensions showing the most progress were governance and workforce. Every organization undergoing evaluation intends to utilize information technology to enhance its management processes. Developing interoperable systems is essential for providing access to information and implementing digital platforms to engage users. However, much work must be done to prioritize person-centered health, enhance predictive analysis, involve users more in decision-making, and improve access to information. Data should be effectively used to create relevant disease planning and prevention information.

In our study, we found similar results regarding the low level of implementation of DT in the health sector. In a study conducted by Woods *et al*. (21) in 2022, the capacity of DT in hospital centers in Australia was evaluated, obtaining an average score of greater than 100 out of 400 points, with a mean of 161/400. This score falls below the general average of DT, which is 200/400. In developing countries such as Peru, private health centers specializing in maxillofacial radiology were assessed, revealing a poor reality in terms of DT. The progress in DT was found to be as low as 13%, highlighting the gap between developed and developing countries. However, even in rural areas of developed countries, DT implementation was found to be greater than 15%. The study by Woods (21) also compared hospital centers in the capital and rural areas, finding a significant association. In contrast, our study compared different centers in different areas of Lima and found no relationship, indicating that the level of implementation is not influenced by the location of the center despite the division of Lima into zones based on per capita growth. When comparing each measurement indicator, the study by Woods showed that the scores of each dimension exceeded those of our study by 50%.

In 2022 Al-Kahtani *et al*. (22) conducted a similar study in public and private hospital health centers in Saudi Arabia. Their results on DHI were similar to those of Woods (21), albeit lower on average. Their results exceeded our study by more than 50% in each dimension evaluated. However, they did not find any differences between the public and private sectors, indicating that the level of DT implementation in the public and private sectors in Saudi Arabia is similar. In our study, DT in radiology centers in different areas of Lima showed similar conditions; that is, well below the average implementation in developed countries. This demonstrates a clear downward trend and a status of being in initial stages in terms of levels of DT implementation in the health sector. The directors of the radiological centers should consider all of this information to initiate changes in this situation, pursuing better prospects and benefits for all involved.

## Conclusions

Maxillofacial radiology centers in Lima are at an initial level of DT. Radiology centers should seriously consider DT to have access to relevant information related to diagnostic and treatment decision-making and to provide better preventive health policies to benefit the population.

## Figures and Tables

**Table 1:** Average values according to DHI dimensions in the different areas of Lima.

Indicators	West		East		Center		North		South		P-value
	Mean	SD	Mean	SD	Mean	SD	Mean	SD	Mean	SD	
DHI	74.19^a^	58.46	50.73^a^	24.23	59.33^a^	23.99	56.29^a^	50.18	43.2^a^	16.42	0.616
Interoperability	29.19^a^	23.58	18.64^a^	7.87	25.50^a^	10.67	24.57^a^	23.80	16.6^a^	10.41	0.615
People-enabled health	21.19^a^	20.31	17.09^a^	11.23	17.67^a^	8.17	17.86^a^	20.07	17.00^a^	9.90	0.930
Predictive analysis	22.88^a^	16.65	14.00^a^	8.15	14.33^a^	11.62	11.57^a^	13.99	8.20^a^	4.97	0.139
Governance and Workforce	30.13^a^	24.35	17.55^a^	8.40	24.50^a^	11.74	23.57^a^	22.83	13.60^a^	10.41	0.198

Similar letters: There are no significant differences between geographic areas. DHI: digital health indicator; SD: standard deviation

## Data Availability

The datasets used and/or analyzed during the current study are available from the corresponding author.
